# Supplementary material for: Association of the past epidemic of Mycobacterium tuberculosis with mortality and incidence of COVID-19
Source: PLoS One. 2021 Jun 18;16(6):e0253169. doi: 10.1371/journal.pone.0253169 (PMC8213125; doi:10.1371/journal.pone.0253169)
Supplement: S1 Fig — (PDF) [file pone.0253169.s001.pdf]

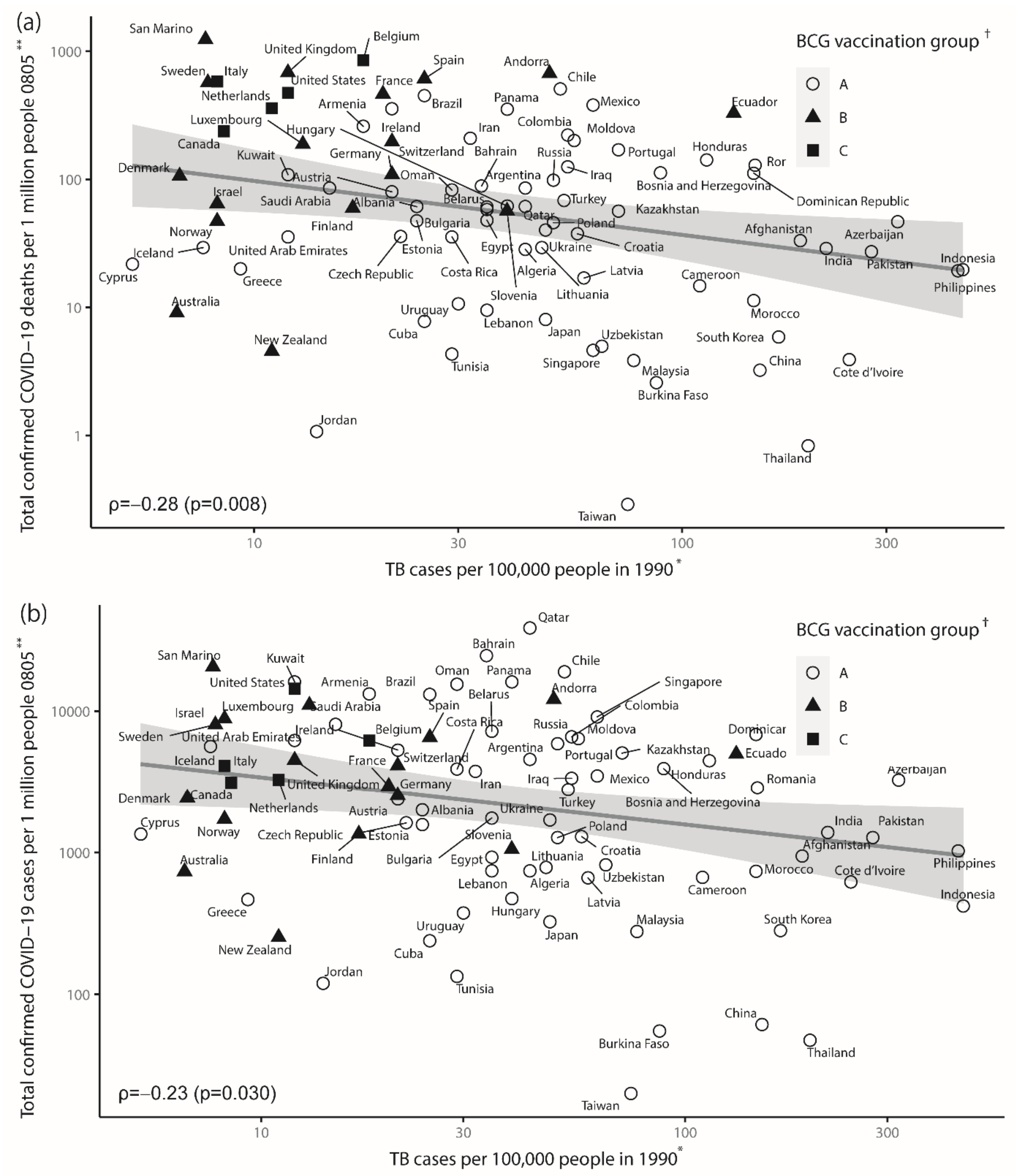

S1 Fig. Scatterplot of the incidence rate of tuberculosis (TB) in 1990 versus (a) the cumulative mortality rate of COVID-19 and (b) cumulative incidence rate of COVID-19 on 5 August 2020 according to BCG vaccination status among 90 countries

*Information was obtained from "Global Note" [12].

**On 5 August 2020. Information was obtained from "Our World in Data" [1].

† Information was obtained from the "BCG World Atlas" [18]. A: The country currently has a universal BCG vaccination programme. B: The country used to recommend BCG vaccination for everyone, but currently, it does not. C: The country never had universal BCG vaccination programmes. Norway was moved from groups A to B [19].

TB, tuberculosis.
