# Supplementary material for: Association of the past epidemic of Mycobacterium tuberculosis with mortality and incidence of COVID-19
Source: PLoS One. 2021 Jun 18;16(6):e0253169. doi: 10.1371/journal.pone.0253169 (PMC8213125; doi:10.1371/journal.pone.0253169)
Supplement: S1 Table — (PDF) [file pone.0253169.s003.pdf]

S1 Table. Association of the decrease in the incidence rate of tuberculosis (per 100,000 people) in 1990 and the mortality rate of tuberculosis (per 100,000 people) in 1950 on the cumulative deaths and cases of COVID-19 (per 1 million people) on 5 August 2020.

	TB incidence in 1990		TB mortality in 1950	
	**Rate ratio [a]**	**(95% CI)**	**Rate ratio [a]**	**(95% CI)**
**COVID-19 mortality**				
Crude model	1.44	(1.07–1.94)	1.39	(0.82–2.38)
Adjusted model 1	1.30	(0.97–1.74)	1.40	(0.82–2.40)
Adjusted model 2	1.00	(0.80–1.24)	9.39	(0.93–95.02)
Adjusted model 3	1.08	(0.84–1.38)	9.32	(0.18–493.74)
**COVID-19 incidence**				
Crude model	1.17	(0.91–1.51)	1.31	(0.76–2.25)
Adjusted model 1	1.33	(1.06–1.67)	1.35	(0.77–2.36)
Adjusted model 2	0.94	(0.77–1.15)	1.14	(0.71–1.82)
Adjusted model 3	0.96	(0.81–1.15)	1.25	(0.74–2.11)

CI, confidence interval; TB, tuberculosis

Adjusted model 1 was adjusted for the prevalence of aged 65 older.

Adjusted model 2 was adjusted for the prevalence of aged 65 older and the prevalence of chronic disease (diabetes and cardiovascular disease).

Adjusted model 3 was adjusted for the prevalence of aged 65 older, prevalence rate of chronic disease (diabetes and cardiovascular disease), and the GDP per capita.

[a] Rate ratio per one unit decrease in the incidence rate of tuberculosis in 1990 and mortality rate of tuberculosis in 1950 (per 100,000 people), respectively.
