# Supplementary material for: Association of the past epidemic of Mycobacterium tuberculosis with mortality and incidence of COVID-19
Source: PLoS One. 2021 Jun 18;16(6):e0253169. doi: 10.1371/journal.pone.0253169 (PMC8213125; doi:10.1371/journal.pone.0253169)
Supplement: Attachment — Submitted filename: POne_0-3_R1_COVID19-TB_Response to Reviewers comments.docx [file pone.0253169.s004.docx]

**Responses to comments from reviewer #1**

We much appreciate your kind and thoughtful comments on our manuscript. We have carefully revised it following your helpful suggestions. Also, following other reviewer’s comment, we have added the results which evaluated the association of the past epidemic of *M. tuberculosis* with the COVID-19 incidence rate in Fig 3, Fig 4 and Table 1. In addition to reviewers comments, we have corrected typos error in the manuscripts and received native English editing service again. We have indicated changes or additions to the main text ('Revised Manuscript with Track Changes') in red. We present our responses to your comments in bold and italic below following your comments.

***Reviewer #1: In this study, Inoue and Kashima analyze the relationship between latent tuberculosis and COVID19 mortality. An inverse association between BCG and COVID19 has been proposed by several ecological and epidemiological studies, while latent tuberculosis was much less studied. From this perspective, this is an useful survey of the data in various countries. However, such ecological studies are prone with many biases, and this needs to be very clearly stated.***

**Reply:**

Thank you very much for your valuable comments. We have revised the manuscript by properly reflecting all your comments, as responded below one by one.

***Comments***

***1.*** ***The authors have tried to correct for age, (some) co-morbidities and GDP, but of course*** ***many biases remain in such studies,*** ***such as (but not restricted to) other co-morbidities, differences in diagnostic and treatment of disease, differences in reporting of COVID19, genetic differences in the population, etc.***

**Reply:**

Thank you for pointing out this. We agree with your comments. We should continuously consider those other country-level differences. Following your comment, we have added this point in the discussion section as followed:

(Page16 line 383–389)

In addition, although we have taken into account the rate of elderly people aged 65 years, country-level chronic disease prevalence (diabetes and coronary heart disease), and GDP per capita, other biases such as other comorbidities, differences in diagnostic and treatment of disease, differences in reporting of COVID-19, and genetic differences in the population remain unadjusted. Our hypothesis needs to be tested continuously and, in the future, examined in different stages and settings of the COVID-19 pandemic.

***2. The authors hypothesize that latent TB induces continuous trained immunity, that may explain the lower mortality due to COVID19. However, a recent study (Khan et al, Cell 2020) demonstrated that TB (in contrast to BCG) actually inhibits trained immunity, and this is counterintuitive for the hypothesis of this study. This aspect needs to be discussed.***

**Reply:**

Thank you very much for your valuable comment. We have read the Khan et al., Cell 2020. Then, we have added a discussion related to this paper in the discussion section as followed:

(Page 15 line 354–360)

A recent animal study [26] demonstrated that *M. tuberculosis* inhibited trained immunity in contrast to BCG, which appeared to contradict our hypothesis. They experimentally administered *M. tuberculosis* to mice intravenously, which resulted in the radical systemic invasion. In fact, all *M. tuberculosis-*administered mice succumbed by 120 days. In such a special situation, it is not surprising that *M. tuberculosis* is life-threatening, including in its effects disrupted immune function. Therefore, their findings may not be directly discussed or compared to ours.

***3.*** ***While large parts of the world are exposed to TB and others not (such as developed countries), everyone in the world is continuously exposed to environmental*** ***non-tuberculous mycobacteria.*** ***Why are these non-tuberculous mycobacteria not protective as well?***

**Reply:**

Thank you for this comment. Our study suggests that the pre-existing immune response against *M. tuberculosis* has the potential to mitigate the disease manifestations from COVID-19. Also, one previous study reported that pre-existing immune responses against human coronaviruses for the seasonal “common cold” mitigate the severity of COVID-19. As you commented, pre-existing immune protection, which is not only against *M. tuberculosis* but also other mycobacteria, might play an important role to protect from COVID-19. Further studies are needed to evaluate the role of pre-existing immune protection. We have also discussed this point in the discussion section. So, we have not added new discussion in this manuscripts, but your advice will college our future study.

(Page 14 line 330–335)

Furthermore, one study reported that pre-existing immune responses against human coronaviruses for the seasonal “common cold” mitigate the severity of COVID-19 [23]. In addition to their finding, this study suggests that the pre-existing immune response against *M. tuberculosis* also has the potential to mitigate the disease manifestations from COVID-19. Further studies are needed to evaluate the role of pre-existing immune protection .

***4.*** ***In some countries the TB-related morbidity and mortality significantly changed during our lifetime. Is that associated with different mortalities in those segments of the population?***

**Reply:**

Thank you for your comment. Following your comment, we have added some sentences in the discussion section as followed:

(Page 15, line 347–354)

Another fact to be noted is that the infection pattern (morbidity and mortality) of tuberculosis has significantly changed in some countries, which might affect the strength of people's training immunity with *M. tuberculosis*. Accordingly, this may be associated with the different mortalities of COVID-19 on the population in those regions to some extent. However, most deaths from COVID-19 are older people who may have been exposed to tuberculosis at a young age. Therefore, this study primarily examined the association between past exposure to tuberculosis and COVID-19 mortality. We hypothesized that latent tuberculosis infection induces continuously trained immunity, which may explain the lower mortality due to COVID-19 found in this study.

We thank the editor and the reviewers again for their helpful comments, which we feel have helped greatly improve our manuscript. We hope that with these modifications, our paper is now acceptable for publication.

Sincerely yours,

**Responses to comments from reviewer #2**

We much appreciate your kind and thoughtful comments on our manuscript. We have carefully revised it following your helpful suggestions. Also, following your comment, we have added the results which evaluated the association of the past epidemic of *M. tuberculosis* with the COVID-19 incidence rate in Fig 3, Fig 4 and Table 1. In addition to reviewers comments, we have corrected typos error in the manuscripts and received native English editing service again. We have indicated changes or additions to the main text ('Revised Manuscript with Track Changes') in red. We present our responses to your comments in bold and italic below following your comments.

***Reviewer #2: In this manuscript, the authors hypothesized the past tuberculosis epidemics may be one of the latent explanatory factors which can reduce the COVID-19 mortality. And they also revealed the BCG vaccination which could modulate or train natural immunity and protect COVID-19 severity as spurious association with regional tuberculosis epidemics .***

**Reply:**

Thank you very much for your valuable comments. We have revised the manuscript by properly reflecting all your comments, as responded below one by one.

***Specific recommendations for revision***

***a)Major:***

***Recently the reverse relation of national or regional tuberculosis incidence and BCG coverage with COVID-19 incidence (M.Madan, S.Pahuaja, A.Mohan, et.al https://doi.org/10.1016/j.puhe.2020.05.042) was found and other researchers mention that BCG can reduce the incidence of COVID-19 (Melvin Joy, B. Malavika, Edwin Sam Asirvatham et.al(https://doi.org.10.1013/jcegh.2020.08.015) The authors might mention about the association of past tuberculosis epidemic and COVID-19 not only mortality but also incidence. And the authors could show the spurious relationship of BCG vaccination and trained natural immunity against COVID-19 via past epidemic of tuberculosis.***

**Reply:**

Thank you for your valuable comment. First of all, we have added M. Joy et al., paper in the introduction and discussion section (Page3, line 49–50 and Page 13, line 310). Regarding the paper by Madan et al., the results of the paper partially supported our hypotheses. So, we have added some sentences in the discussion section as followed:

(Page13–14, line 315–321)

In addition, one previous study reported that current high-tuberculosis-burden countries had a lower incidence of COVID-19, irrespective of the BCG vaccine status of the country, and focused on cross-immunity between *Mycobacterium* species and COVID-19 [22]. This study roughly categorized the examined countries into four groups, which are the combination of tuberculosis high and low incidence and BCG high and low coverage. Although it did not evaluate the past burden of tuberculosis, the findings may be partially consistent with ours.

Regarding with incident of COVID-19, we have evaluated the association of the past tuberculosis epidemic with the incidence of COVID-19, and have added the result in the main text, Fig 3, Fig 4, and Table 1. These results of COVID-19 incidence would also strongly support our hypothesis. We have also amended the title of this manuscript as followed:

(Title)

Association of the past epidemic of *Mycobacterium tuberculosis* with mortality and incidence of COVID-19

***b)Minor***

***2-2. Page2 line17 should be written precisely as*** ***The coronavirus disease 2019(COVID-19)*** ***caused by SARS-COV2***

**Reply:**

We have amended the term in the abstract and the introduction as follows:

(Page 2, line 17–19)

The coronavirus disease 2019 (COVID-19) pandemic, caused by the severe acute respiratory syndrome coronavirus 2 (SARS-CoV-2), has created a remarkable and varying impact in every country, inciting calls for broad attention.

(Page 3, line 41–43)

The ongoing novel coronavirus disease 2019 (COVID-19) pandemic, precipitated by the severe acute respiratory syndrome coronavirus 2 (SARS-CoV-2), while a major concern, remains a mystery that puzzles the world.

***Page2 line18*** ***each region may be written each region*** ***on earth***

**Reply:**

Thank you for pointing out this. We reconsider this term. The term “country” is more suitable to represent the current situation than “region”. Thus, we have amended the sentence as follows:

(Page 2, line 17–19)

The coronavirus disease 2019 (COVID-19) pandemic, caused by the severe acute respiratory syndrome coronavirus 2 (SARS-CoV-2), has created a remarkable and varying impact in every country, inciting calls for broad attention.

(Page 4, line 43–44)

One part of the enigma is the remarkable and varying impact on mortality and incidence in each country.

***Page2 line19 may paraphrase to explain the difference in mortality***

**Reply:**

Following your above comment, we have added the results of the COVID-19 incidence. Thus, we have amended the sentence as follows:

(Page 2, line 20–22)

Herein, we hypothesised that the past epidemic of *Mycobacterium tuberculosis* may act as a latent explanatory factor for the worldwide differences seen in COVID-19 impact on mortality and incidence.

***Page2 line30*** ***trained immunity may rewrite trained natural immunity***

**Reply:**

Thank you for pointing out this. We have discussed the term ‘train immunity’ again. In our context, the term “trained immunity” is more suitable to use instead of “trained natural immunity” So, we keet the term “trained immunity” and we have replaced ‘train natural immunity’ with ‘trained immunity in the text and Fig 1.

***Page3 line51 new-born may be*** ***newborn***

**Reply:**

We reconsider the term “newborn”. The term “neonates” is more suitable to use than “newborn” because we have used “infants” in the same sentence. Thus, we have replaced “new-born” with “neonates” on Page 3 line 56.

***Page5 line 102 ditto***

**Reply:**

We have replaced “newborn” with “neonates” on Page 5 line 111.

***Page15 line344 immune may be i******mmunity***

**Reply:**

We have replaced “immune” with “immunity” on Page 17 line 395

We thank the editor and the reviewers again for their helpful comments, which we feel have helped greatly improve our manuscript. We hope that with these modifications, our paper is now acceptable for publication.

Sincerely yours,
